# How we mitigated and contained the COVID-19 outbreak in a hemodialysis center: Lessons and experience

**DOI:** 10.1017/ice.2020.161

**Published:** 2020-04-23

**Authors:** Ke Su, Yiqiong Ma, Yujuan Wang, Yuan Song, Xifen Lv, Zhongping Wei, Ming Shi, Guohua Ding, Bo Shen, Huiming Wang

**Affiliations:** 1 Department of Nephrology, Renmin Hospital of Wuhan University, Wuhan, Hubei, PR China; 2 Department of Cardiology, Renmin Hospital of Wuhan University, Wuhan, Hubei, PR China

*To the Editor*—COVID-19 has become a worldwide pandemic. After 2 months of strict control and prevention measures, the COVID-19 epidemic has been contained successfully in Wuhan. We have summarized lessons and experiences related to the reduction of nosocomial COVID-19 in the hemodialysis center for the benefit of healthcare providers and administrations outside China who are facing the challenges of the COVID-19 pandemic.

Hemodialysis patients are particularly vulnerable to infection and may exhibit greater variations in clinical symptoms and infectivity. Hemodialysis patients are susceptible to infection for the following reasons: (1) Hemodialysis patients require frequent transportation to and from the hospital and their residence to receive dialysis 2 to 3 times per week, which increases the risk of COVID-19 transmission. (2) Hemodialysis patients often require care from family members or caregivers, and if a caregiver is infected, they can transmit the virus to all close contacts, including the hemodialysis patient. (3) The hemodialysis center is a relatively open space with personnel (eg, medical staff and facility workers), patients, and their family members. Thus, many people gather in hemodialysis centers, posing a risk for a virus transmission cluster. (4) Hemodialysis patients infected with COVID-19 may lack typical clinical symptoms (eg, fever, cough, or other respiratory symptoms, or the typical ground-glass image computed tomography (CT) scan of the lungs); they may appear asymptomatic or may have mild symptoms. Chest CT images of hemodialysis patients often show acute exudative lesions, lung consolidation, or interstitial changes. These factors increase the difficulty medical workers face in identifying and diagnosing COVID-19 in hemodialysis patients. For these reasons, SARS-CoV-2 spreads quickly in hemodialysis centers.

To prevent the spread of SARS-CoV-2 in our hemodialysis center, the People’s Hospital of Wuhan University, we consulted with the Hubei Province public health authorities to develop a series of key strategies to help prevent and mitigate the spread of SARS-CoV-2. Here, we describe the control measures that were implemented.

## Repeat and constant screening for infected patients

To maximize the safety of hemodialysis patients and staff, we continually monitored all persons in our hemodialysis center, including hemodialysis patients and their family members (or caregivers), healthcare workers, and facility workers. We recommend monitoring body temperature and respiratory symptoms, conducting routine blood routine tests, conducting nasopharyngeal or pharyngeal swab SARS-CoV-2 nucleic acid tests, and performing chest CT scans to screening for COVID-19.^1^ Testing for SARS-CoV-2 IgG and IgM antibodies in serum is also recommended.^2^ Chest CT scans are very important in screening hemodialysis patients for COVID-19. We repeated CT scans every 2 weeks to recognize and isolated patients as early as possible in the incubation period.

**Fig. 1.**
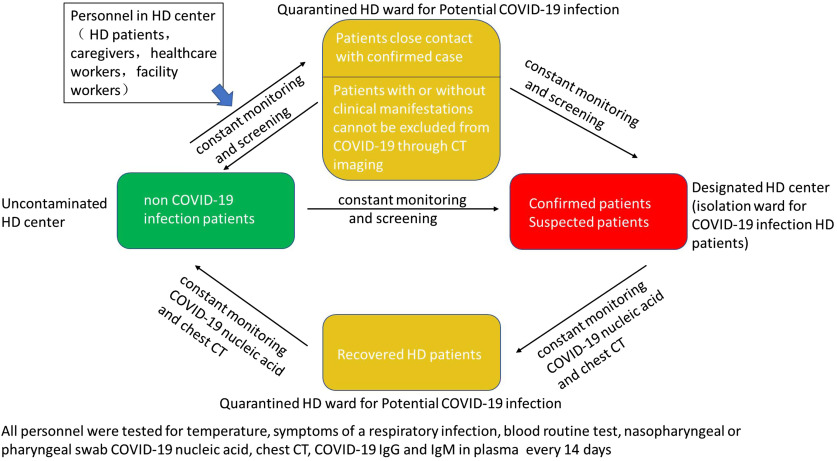
Personnel cohort in our hemodialysis center and distribution to a different ward .

## Evaluation and classification on each person by epidemic situation

Based on screening results and the Guidelines of the China National Health Commission (6th and 7th editions), personnel in our hemodialysis center can be classified into 5 groups: (1) confirmed cases: a person with laboratory confirmation of COVID-19 infection (COVID-19 nucleic acid testing positive), irrespective of clinical signs and symptoms; (2) suspected cases: patients who satisfy epidemiological and clinical criteria (fever or respiratory symptoms and typical CT imaging features) but without laboratory confirmation; (3) patients with clinical manifestations but who cannot be excluded from COVID-19 through CT imaging; (4) those who have had close contact with a confirmed case; and (5) non–COVID-19 patients.

## Allocation and circulation between designated facilities

We distributed hemodialysis patients to different hemodialysis centers or hospitals according to the screening results as follows (Fig. 1):

(1) Hemodialysis patients with confirmed or suspected COVID-19 infection were required to be admitted to a negative pressure isolation ward of specified hospitals where only hemodialysis patients with COVID-19 were cared for. If the capacity of the isolation facility was overloaded, the “fixed dialysis care model” outlined below was followed.^3^
(2) Patients who were not SARS-Cov-2 positive continued hemodialysis at the original uncontaminated hemodialysis center.
(3) Hemodialysis patients with clinical manifestations but who could not be excluded from COVID-19 through CT imaging and who had had due to close contact with a confirmed case remained hospitalized in a quarantined ward and received continuous renal replacement therapy (CRRT). Dialysis shifts, dialysis units, and caregiver staff were not be changed to prevent cross contamination and infection. Contact with relatives was minimized. These quarantine hemodialysis patients underwent the testing outlined previously during the 14-day quarantine period. Once a hemodialysis patient converted to a confirmed case, the patient was treated under confirmed case management protocols. Screening for patients only once was not enough; repeated screening was needed to identify probable cases.
(4) When hemodialysis patients with COVID-19 recovered, they were transferred to a quarantine ward for recovered patients for 14 days of observation. After 2 negative nucleic acid tests, the patient could be transferred to the uncontaminated hemodialysis center. If any healthcare personnel were confirmed with COVID-19 or had a probable case, they were also quarantined.^4^

These measures have proven effective. After the beginning of outbreak, there were 37 COVID-19 cases among 230 hemodialysis patients (16.09%) and 4 cases among 33 staff (12.12%) who were suspected cases. Furthermore, 7 confirmed and suspected hemodialysis patients died between February 4 and February 13, 2020.^5^ Also, 5 confirmed patients and no healthcare workers or facility workers were infected between February 14 and March 1, 2020. Collectively, these strategies can effectively minimize clusters of infection while providing timely treatment for hemodialysis patients.
